# Targeting STAT3 Signaling Pathway in Colorectal Cancer

**DOI:** 10.3390/biomedicines9081016

**Published:** 2021-08-15

**Authors:** Antonios N. Gargalionis, Kostas A. Papavassiliou, Athanasios G. Papavassiliou

**Affiliations:** 1 Department of Biological Chemistry, Medical School, National and Kapodistrian University of Athens, 11527 Athens, Greece; agargal@med.uoa.gr (A.N.G.); konpapav@med.uoa.gr (K.A.P.); 2 Department of Biopathology, Aeginition Hospital, Medical School, National and Kapodistrian University of Athens, 11528 Athens, Greece

**Keywords:** STAT3, colorectal cancer, colorectal cancer inflammation, colorectal cancer immunotherapy, colorectal cancer resistance

## Abstract

Signal transducer and activator of transcription 3 (STAT3) is a critical transcription factor that has been firmly associated with colorectal cancer (CRC) initiation and development. STAT3 mediates key inflammatory mechanisms in colitis-associated cancer, becomes excessively activated in CRC, and enhances cancer cell proliferation, tumor growth, angiogenesis, invasion, and migration. STAT3 hyperactivation in malignant cells, surrounding immune cells and cancer-associated fibroblasts, mediates inhibition of the innate and adaptive immunity of the tumor microenvironment, and, therefore, tumor evasion from the immune system. These features highlight STAT3 as a promising therapeutic target; however, the mechanisms underlying these features have not been fully elucidated yet and STAT3 inhibitors have not reached the clinic in everyday practice. In the present article, we review the STAT3 signaling network in CRC and highlight the current notion for the design of STAT3-focused treatment approaches. We also discuss recent breakthroughs in combination immunotherapy regimens containing STAT3 inhibitors, therefore providing a new perception for the clinical application of STAT3 in CRC.

## 1. Introduction

Colorectal cancer (CRC) represents one of the leading types of malignancy, in both genders, worldwide. Several risk factors have been acknowledged, such as increasing age and positive family history (hereditary CRC syndrome accounts for 5–7% of the patients), as well as lifestyle factors, featuring obesity, smoking, and increased intake of alcohol and red/processed meat [1]. Most CRCs initiate from cancer stem cells (CSC)s inside the colonic epithelium, accumulating progressive genetic and epigenetic alterations. These alterations lead to impaired gene expression and/or function, therefore favoring the activation of oncogenes and the downregulation of tumor suppressor genes [2].

Extensive research, regarding CRC molecular pathogenesis, has reached a well-defined molecular classification of the disease, which encloses the following four distinct molecular subtypes: (1) the canonical subtype, characterized by high somatic copy number alterations, (2) the microsatellite instability immune subtype, (3) the metabolic, and (4) the mesenchymal subtype [3]. CRC treatment options include endoscopic and surgical treatment, radiotherapy for rectal cancers, local therapies for metastatic disease, systemic chemotherapy, and novel targeted agents and immunotherapy [1]. Specifically, targeted therapy and immunotherapy have aided in the configuration of more efficient patient selection criteria, regarding CRC treatment options, therefore offering prolonged survival and increased progression-free survival for certain patient groups with distinct molecular characteristics [4].

In this context, transcription factors (TFs)—once referred to as “undruggable”— represent a unique class of drug targets. TFs are proteins that integrate the signal transduction of critical signaling networks in health and disease. They have the capacity to bind to the DNA of the promoter or enhancer regions of specific genes, and consequently regulate the respective gene transcription. Targeting TFs in cancer can block epithelial-to-mesenchymal transition (EMT), immune evasion, stem cell properties, impaired differentiation and/or cell death, and disrupt autoregulatory circuits that drive cancer [5]. In CRC, genetic and epigenetic alterations lead to hyperactivation and oncogenic function of several chief TFs, including signal transducers and activators of transcription (STATs). On the other hand, TFs such as p53, runt-related transcription factor 3 (RUNX3), forkhead box O3 (FOXO3), and Kruppel-like factor 4 (KLF4), are inactivated, and thus were prevented from exerting their tumor-suppressive functions [6].

Signal transducer and activator of transcription 3 (STAT3) is a member of the STAT family of cytoplasmic TFs, and is also the family member that has been most associated with CRC initiation and development [7]. STAT3 is the point of integration for various tumorigenic signaling pathways and regulates the immune response against tumors. Notably, STAT3 depicts a unique feature in CRC, as the interleukin-6 (IL-6)/STAT3 signaling axis represents a key inflammatory mechanism in colitis-associated cancer (CAC) [8]. STAT3 is also abundantly activated in CRC and enhances cancer cell proliferation, tumor growth, invasion, and migration [9]. These features highlight STAT3 as a promising therapeutic target; however, the mechanisms underlying these features have not been fully elucidated yet, in order for STAT3 inhibitors to reach the clinic [10]. In the present article, we review the STAT3 signaling network in CRC and highlight the current status for the development of STAT3-targeting treatment approaches. We also discuss recent breakthroughs in combination immunotherapy regimens containing STAT3 inhibitors, offering new insight into the translational application of STAT3 in CRC [11].

## 2. STAT3 Structure and Regulation of Activity in Normal Cells and Disease

The STAT family consists of the following seven structurally and functionally associated proteins: STAT1, STAT2, STAT3, STAT4, STAT5a and STAT5b, and STAT6. STATs function as cytoplasmic signal transducers and regulators of gene transcription in the nucleus. These molecular events occur following cytokine and growth factor stimuli, and they are critical for the regulation of innate and acquired host immune responses [12,13].

**Figure 1:**
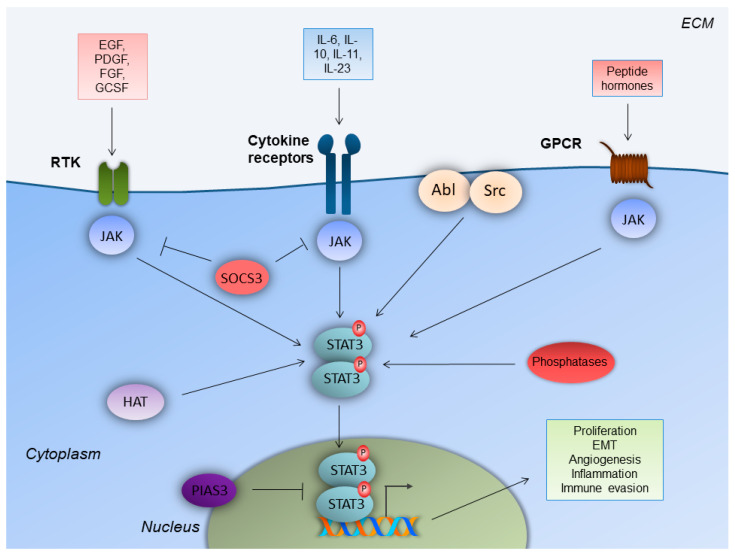
STAT3 signaling in colorectal cancer cells. Upon ligand binding, receptor tyrosine kinases (RTK), cytokine receptors and G-protein-coupled receptors (GPCR) lead to Janus kinase (JAK) recruitment, which facilitates signal transducer and activator of transcription-3 (STAT3) phosphorylation and homodimerization. STAT3 homodimers enter the nucleus and enhance gene transcription to induce colorectal cancer cell-specific properties. Regulation of STAT3 is achieved by non-receptor tyrosine kinases (Src, Abl), histone acetyltransferases (HAT), tyrosine phosphatases and E3 SUMO-protein ligase (PIAS3). Epidermal growth factor receptor (EGFR); extracellular matrix (ECM); fibroblast growth factor receptor (FGFR); granulocyte colony-stimulating factor receptor (GCSFR); platelet-derived growth factor receptor (PDGFR); suppressor of cytokine signaling 3 (SOCS3).

STAT3 was firstly identified as a component of IL-6 signaling, which co-immunoprecipitates as a phosphoprotein in a complex with acute phase response factor (APRF) in HepG2 hepatoma cells [14]. It is an 88-kDa protein that harbors a conserved Src homology 2 (SH2) domain within its primary amino acid sequence [15]. Cytokines bind to classic cytokine receptors (e.g., IL-10), and membrane-bound and soluble receptors (e.g. IL-6, IL-11, and IL-23). Growth factors bind to their respective growth factor receptors, and peptide hormones bind to their respective G-protein-coupled receptors (GPCR)s (Figure 1) [15,16]. Upon ligand binding, the receptors mainly activate the Janus kinase (JAK) family of kinases, which trans-phosphorylate each other. JAKs further phosphorylate the C-terminus of the receptor subunit. The latter event attracts STAT3, with its SH2 domain, to a related docking site in the cytoplasmic tail of the activated receptor subunit. This leads to STAT3 phosphorylation on the Y705 tyrosine residue. 

STAT3 is also phosphorylated by other intracellular kinases, such as the non-receptor tyrosine kinases Src and Abl. STAT3 phosphorylation leads to its homodimerization. The homodimer enters the nucleus, in order to exert its transcriptional activity regarding genes with diverse biological consequences [16]. STAT3 acetylation, by histone acetyltransferase on a lysine residue (K685), is an alternative mechanism of STAT3 activation that stabilizes the STAT3 dimer [17]. On the other hand, STAT3 negative regulation is mainly controlled by tyrosine phosphatases, which dephosphorylate/deactivate STAT3. STAT3 is inhibited by a suppressor of cytokine signaling 3 (SOCS3) at the receptor level, and by E3 SUMO-protein ligase (PIAS3) at the transcriptional level (Figure 1) [17,18,19,20,21].

STAT3 activity is implicated in a number of physiological processes, including cell proliferation, apoptosis, differentiation, production of mature blood cells, and regulation of the immune system [22]. Specifically, STAT3 plays a critical role in the development of CD4+ T helper 2 (Th2) cells [23,24], and T cells that produce IL-17 (Th17 cells), by regulating the transcription of the Th17 lineage-specific TFs [25,26,27]. STAT3 also regulates the activity of CD4+ regulatory T cells (Tregs), by physically associating with their lineage-specific TF, forkhead box P3 (FOXP3) [28]. STAT3 is critical in innate immunity, IL-6 signaling, and the acute phase response. Furthermore, it serves key functions in neutrophil and macrophage development, but also holds pro- and anti-inflammatory properties [29].

## 3. Mechanisms of STAT3 Implication in Tumorigenesis

STAT3 signaling dysregulation has been associated with various distinct clinical entities. Hyper-IgE syndrome has been attributed to *STAT3* germline loss-of-function mutations. Lymphocytic leukemia and multi-organ autoimmunity are caused by germline and somatic gain-of-function mutations, respectively [27]. Aberrant STAT3 regulation has been related with autoimmune, inflammatory disorders, such as rheumatoid arthritis, multiple sclerosis, and inflammatory bowel disease [22], as well as with the pathobiology of advanced glycation end products (AGEs) in polycystic ovary syndrome (PCOS) [30,31].

The canonical STAT3 pathway that functions transiently and is strictly regulated in normal cells, has been documented to be persistently activated in a variety of solid and hematological malignancies [32]. STAT3 is also activated by non-canonical pathways in tumorigenesis, which involve STAT3 phosphorylation in a (Ser)727 alternative site of the C-terminus, by mitogen-activated protein kinase (MAPK), c-Jun N-terminal kinase (JNK), or protein kinase C (PKC) pathways. Constitutively activated STAT3 translocates to the nucleus and enhances gene transcription, which favors cell proliferation, resistance to apoptosis, survival, angiogenesis, tumor-promoting inflammation, tumor-mediated immune evasion, and, ultimately, invasion and metastasis. Recent evidence demonstrates that STAT3 promotes tumorigenesis through additional processes, including inflammation-promoting carcinogenesis, obesity and/or metabolism, CSCs, and pre-metastatic niche formation [33].

Aberrant STAT3 activation is mainly attributed to the excessive production of cytokines and growth factors in the tumor microenvironment (TM), high expression of protein tyrosine kinases, and epigenetic suppression of negative regulators of STAT3, such as SOCS3 and protein tyrosine phosphatases [34]. Particularly, the activation of the IL-6/JAK/STAT3 core axis is observed in many types of cancer. IL-6 is overexpressed in the TM and stimulates the JAK/STAT3 pathway, which is a molecular event that is associated with poor prognosis for cancer patients. JAK2 is specifically activated in myeloproliferative disorders and activates STAT3 [35]. IL-6 plays a protective role for cancer cells, against DNA damage, apoptosis, and oxidative stress [36]. Regarding angiogenesis, STAT3 is activated in cancer cells by reactive oxygen species (ROS), mechanistic target of rapamycin complex 1 (mTORC1) and/or IL-6, and further induces hypoxia-inducible factor 1-alpha (HIF-1a) activation during hypoxia. HIF-1a induces the transcription of vascular endothelial growth factor (VEGF), which, in turn, affects the surrounding endothelial cells. This event activates, once again, STAT3 via the VEGF receptor (VEGFR). In endothelial cells, STAT3, along with HIF-1a and sp1, promotes the transcription of genes that mediate the cell growth, survival, and migration of endothelial cells, therefore promoting angiogenesis [37].

STAT3 represents an attractive therapeutic target, because it coordinates the crosstalk between cancer cells and immune cells from the TM, thus regulating the anti-tumor immune response [38]. This function is mediated by an IL-6/STAT3-positive feedforward loop that augments STAT3 activity [38]. Therefore, by inhibiting STAT3, such anti-tumor immune response could be alleviated.

## 4. Mechanisms of STAT3 Engagement in Colorectal Tumorigenesis

### 4.1. STAT3 Expression and Regulation in Colorectal Cancer Cells

The immunohistochemical evaluation of pSTAT3 in a large dataset of 724 CRCs, showed that pSTAT3 expression is significantly associated with higher mortality and increased lymphoid reactions surrounding the tumors [39]. The immunohistochemical evaluation of p-STAT3 in 108 CRC cases demonstrated cytoplasmic and nuclear expression, positive correlation of pSTAT3 expression, with the depth of the tumor invasion, Dukes, TNM stage, and reduced overall survival [40]. STAT3-enhanced activation in CRC occurs from IL-6 in the serum and the tumor microenvironment, from growth factors and growth factor receptors, tyrosine kinases (Src, Bcr-Abl), and loss-of-function mutations for the STAT3 suppressors/inhibitors (phosphatases, SOCS, PIAS) [35].

Beyond direct STAT3 regulation by upstream molecules, STAT3 is also activated by the suppression of STAT3 negative regulators, such as PTPRT [41]. PTPRT functions as a tumor suppressor gene, and is mutated in colon, lung, stomach, and skin (melanoma) cancers. In CRC, it is the most frequently mutated tyrosine phosphatase among the tyrosine phosphatome that was examined in 18 CRCs. PTPRT presents five mutations that decrease its activity [42,43]. STAT3 has been identified as a substrate of PTPRT. The epigenetic suppression (promoter hypermethylation) of PTPRT occurs in several human cancers, but most notably in CRC (78.7%, 289/367 tumors analyzed) [44].

However, there are data demonstrating that STAT3 is able to elicit different responses according to the type of tumor and the mutational status. Therefore, it may function either as an oncogene or a tumor suppressor gene. For example, STAT3 acts as a tumor suppressor in glioblastoma tumors, with phosphatase and tensin homolog (PTEN) loss-of-function mutations, lung cancer, and thyroid carcinoma [45]. In CRC, it has been demonstrated that STAT3 does not only elicit invasive and metastatic effects, but can also inhibit EMT. EMT inhibition is achieved through downregulation of the EMT-promoting TF Snail, by a glycogen synthase kinase (GSK) 3*β*-mediated degradation in Apc*^Min/+^* mice [46]. Corroborating findings in experiments with conditional ablation of STAT3 (*Stat3^ΔIEC^* Apc*^Min/+^* mice), show that STAT3 is activated during the initial stages of tumor development, but STAT3 negative regulation is needed for CRC tumor invasion [47]. Therefore, the application of STAT3 inhibition should be performed in respect to the pleiotropic functions of this TF. STAT3 modulation should be tailored according to the specific tumor type, in order to avoid promoting cancer invasion.

### 4.2. STAT3 in Invasion and Metastasis

STAT3 is implicated in the invasion and metastasis of CRC cells, through various mechanisms. In CRC cells, the IL-6/STAT3 axis promotes EMT and, therefore, the CRC aggressive phenotype via the STAT3-dependent transcriptional upregulation of Fos-related antigen-1 (Fra-1) TF. Fra-1 belongs to the Fos family of TFs, and is associated with phenotype invasive transformation and EMT in various types of cancer. In a 229 CRC patient cohort, Fra-1 expression was positively correlated with an increased depth of invasion, lymph node, and liver metastases. Fra-1 was also expressed according to IL-6 and pSTAT3 expression. In CRC cells, STAT3 needs to be both phosphorylated and acetylated to bind to the Fra-1 promoter and induce Fra-1 transcription [48].

MiR-34 is known to suppress EMT. The *MiR-34* gene is a target of activated STAT3, which causes transcriptional repression of miR-34, and therefore induces EMT promotion in CRC cells and tumors [49]. Furthermore, the p53-inducible miR-34a control of the tyrosine kinase colony-stimulating factor 1 receptor is alleviated in CRC, through a feedback loop that is mediated by STAT3 [50]. STAT3 is also involved by enhancing the acquisition of EMT and cancer stem cells traits by CRC cells, through transcriptional induction of NANOG TF. NANOG is a homeobox TF that has been found to positively regulate Slug, which is an EMT-promoting TF. In this feedforward transcriptional cascade, insulin growth factor (IGF) signaling increases STAT3-inducing activation, which, in turn, favors NANOG/Slug activity, and promotes cancer cells stemness and EMT [51]. Homeobox protein NANOG, regulated by IL-6, also regulates the enzymes of cytochrome P450 (CYP450), which are implicated in CRC development, through the activation of chemical carcinogens. In particular, IL-6 induces CYP2E1 upregulation in a STAT3-dependent manner, which has been linked to excessive alcohol intake and the potential risk for CRC [52]. In addition, STAT3 phosphorylation mediates the high activity of laminin 521, which belongs to the basement membrane proteins. This feature promotes tumor invasiveness and self-renewal, therefore inducing CRC cell dissemination to the metastatic sites [53].

### 4.3. STAT3 in Angiogenesis

During hypoxia, STAT3 is activated in cancer cells by ROS, mTORC1, and/or IL-6, and further induces HIF-1a. This transcription factor induces the transcription of VEGF. This event affects the surrounding endothelial cells, in order to activate, once again, STAT3 via VEGFR. In endothelial cells, STAT3, along with HIF-1a and sp1, promotes the transcription of genes that mediate cell growth, survival, and migration of ECS, therefore angiogenesis [37]. Corticotrophin-releasing hormone (CRH) and its receptor CRHR contribute to CRC cells proliferation, and promote angiogenesis. CRH and CRHR1 are overexpressed in CRC human tissues and CRC cell lines compared to normal colon cells. The treatment of DLD1 and HCT116 cells with CRH increased STAT3 (Y705) phosphorylation, through the phosphorylation of JAK2, as well as the tube formation of human umbilical vein endothelial cells (HUVECs) in CRH-treated CRC medium. This event occurs by an autocrine positive feedback loop, during which CRH upregulates IL-6 and VEGF via NF-kB. At the same time, CRH becomes the upstream activator of JAK2 and STAT3 [54]. STAT3 promotes angiogenesis in CRC, through an alternative mechanism that implicates CD24. CD24 is a membrane molecule, whose high expression is associated with increased microvascular density. CD24 induces VEGF expression through the activation of heat shock protein 90 (HSP90) and downstream STAT3 [55]. STAT3 is implicated in lymphangiogenesis, which is an early event in CRC progression. BRG1, a member of the epigenetic complex SWI/SNF, co-immunoprecipitates with STAT3 in CRC cells, and has been associated with STAT3 activation. BRG1 presents low expression in human CRC tumors, which are abundant in lymphatic vessels. *BRG1* knockdown activates STAT3 and induces the transcription of VEGFC. Therefore, the BRG1/STAT3/VEGFC axis promotes lymphangiogenesis and lymph node metastases in CRC cells and tumor tissues [56]. The mechanism through which *BRG1* knockdown activates STAT3 is unknown. However, it is possible that BRG1 transcriptional effector is a STAT3 negative regulator and *BRG1* knockdown alleviates such negative modulation, thus activating STAT3.

### 4.4. STAT3 in Tumor-Promoting Inflammation

Chronic inflammation is involved in several processes during tumorigenesis, such as proliferation, survival, transformation, invasion, angiogenesis, and metastasis [57]. Regarding CRC, in particular, patients with inflammatory bowel diseases (ulcerative colitis—Crohn’s disease) have increased risk of developing CRC, and colitis-associated CRC (CAC) presents poor prognosis [57,58]. Studies that have employed gain-of-function and loss-of-function in vivo models show that STAT3 mediates the impact of IL-6 and IL-11 on the initiation and development of CAC. STAT3 activity is considerably reduced in the colon and intestinal epithelial cells of *IL-6^-/-^* mice, whereas STAT3 silencing in intestinal epithelial cells has a profound effect on CRC tumorigenesis in vivo [8]. IL-6 and IL-11 activate STAT3 through gp130, thus establishing an IL-6, IL-11/gp130/STAT3 signaling axis that prevents enterocytes from apoptosis, and facilitates cell survival and cell proliferation, ultimately promoting tumor growth [59].

STAT3 is a critical regulator of inflammatory processes. Therefore, it has also emerged as key molecule for inflammation-associated processes, during tumor development of both CAC and sporadic CRC [60]. STAT3 is a core mediator of CAC, since it facilitates IL-6-induced proliferating and anti-apoptotic effects on intestinal epithelial cells [8]. Furthermore, the status of the gut microbiome and the proportion of certain gut microbiota are also important during inflammation-associated CRC, with certain bacteria either promoting or suppressing tumorigenesis [61,62]. Therefore, the abundance of *Streptococcus bovis/gallolyticus*, enterotoxigenic *Bacteroides fragilis*, and *Escherichia coli* NC101 have been suggested as risk factors for CRC [63]. CRC causes disruption of the epithelial barrier integrity, and therefore immune responses are evoked by the gut microbiota pathogen recognition receptors (PRRs). PRRs recognize pathogen-associated molecular patterns (PAMPs), and inflammatory signaling pathways become activated, ultimately leading to the secretion of proinflammatory cytokines, such as interleukin-1b (IL-1b), IL-6, IL-11, and tumor necrosis factor (TNF). STAT3 becomes activated in colon epithelial cells and induces oncogenic gene transcription that mediates proliferation and survival. PRR signaling is negatively regulated by molecules such as IL-1 receptor-associated kinase-M (IRAK-M) [64]. IRAK-M ensures STAT3 stabilization and regulates the status of the intestinal microbiome. In doing so, IRAK-M promotes cancer cell proliferation and is associated with poor prognosis in CRC [64].

## 5. STAT3 in Colorectal Cancer Treatment

We already know that STAT3 inhibition in vitro suppresses tumor growth and promotes apoptosis in cancer cells. STAT3 integrates the oncogenic signaling of multiple upstream kinase molecules. Therefore, STAT3 inhibition is a promising therapeutic strategy that could abrogate the oncogenic impact of several upstream activated kinase molecules. Although JAK1/2 inhibition has favorable effects in JAK/STAT-driven hematological malignancies, agents that target STAT3 are still in the early phases of development. Strategies targeting STAT3 are categorized into the following groups: (1) inhibitors of the SH2 domain or STAT3 dimerization, (2) upstream tyrosine kinase inhibitors, (3) STAT3 pathway oligonucleotides, (4) inhibitors of STAT3–DNA binding, and (5) mimics of STAT3 negative regulators [32]. There is a number of STAT3 inhibitors with promising efficacy in preclinical studies, which have entered clinical trials (Table 1) [10]. These STAT3 inhibitors include small peptides and molecules, peptide mimics, oligonucleotides, and siRNA anti-STAT3 compounds. Specifically, STAT3 inhibition is mainly focused on the STAT3 coiled-coil, SH2, and DNA-binding domains [10,35].

### 5.1. STAT3 Engagement in Mechanisms of Resistance to Therapy

STAT3 is activated in CRC, among other types of malignancy, and is also found as a regulator of CRC cell resistance to chemoradiotherapy. Specifically, STAT3 is implicated in the mechanisms of resistance to fluorouracil (5FU)-based treatments in conjunction with radiotherapy. STAT3 expression is positively correlated with increased resistance. STAT3 knockdown and inhibition with the small-molecule inhibitor Stattic in the CRC cell lines SW480 and SW837, leads to decreased clonogenic potential, following irradiation and treatment with 5FU. Stattic is a selective inhibitor that does not block STAT1, but binds to the SH2 domain and retains STAT3 to the cytoplasm [65]. The treatment of CRC xenografts with Stattic sensitizes tumors to chemoradiotherapy and significantly delays their growth compared to controls [66]. The IL-6/STAT3 pathway also facilitates resistance to irinotecan, by increasing the metabolism of lactate in hypoxic CRC cells driven by hepatic stellate cells in CRC liver metastatic sites [67]. Abundant IL-22 expression in CRC tumor tissues and patients’ peripheral blood, who received folinic acid (leucovorin) (FOL), fluorouracil (5-FU) (F), and oxaliplatin (Eloxatin) (OX) (FOLFOX) chemotherapy, is associated with resistance to the treatment and decreased survival. These aggravating effects are mediated by IL-22-induced STAT3 activation and the further STAT3-dependent transcriptional increase in IL-8 [68].

LL1 is also a specific inhibitor of STAT3 phosphorylation, blocking dimerization and translocation to the nucleus. In CRC cells, LL1 suppresses tumor growth, colony formation, invasion and metastasis, and tumor growth, in HCT116 CRC xenografts [69]. Bruceantinol is a STAT3 suppressor that blocks the DNA binding of STAT3. In CRC cells and xenografts, bruceantinol suppresses tumor proliferation and tumor growth, but also sensitizes cells to MEK inhibitors, thus surpassing the STAT3 point of resistance [70]. CAFs from the TM secrete factors that induce the exit of CRC cells from mitosis, and confer resistance to oxaliplatin and 5-FU in CRC cells. These ligands are present in CAFs conditioned medium and activate the JAK/STAT pathway. Short hairpin RNA (shRNA)-stable knockdown of STAT3 is able to sensitize cells to oxaliplatin and 5-FU, therefore suggesting that the CAF-associated resistance mechanism is promoted through STAT3 activation [71].

The most clinically efficient STAT3 suppressor is napabucasin (BBI608), and there are several ongoing and completed clinical trials in solid tumors and CRC (Table 1) [72]. Napabucasin is known to favor cancer cell stemness. In CRC, there is a completed clinical trial where napabucasin was tested in 282 patients with advanced CRC. The results of the study showed that there was no difference in the overall survival, but when the patients were stratified, according to positive pSTAT3 expression, they had increased survival [73]. A lipophilic anthraquinone that is found in medicinal plants, termed rhein, inhibits STAT3 activity and inhibits cell proliferation, induces apoptosis and causes cell cycle arrest, in a dose-dependent manner. Rhein and napabucasin could also sensitize CRC cells to the EGFR tyrosine kinase inhibitor (TKI) erlotinib, therefore suggesting a potential synergistic effect of EGFR and STAT3 inhibition in CRC [74].

### 5.2. STAT3 in Combination with Immunotherapy

Aberrant regulation of immune checkpoints is a mechanism via which cancer cells avoid immune destruction, and tumor development is facilitated [11]. Therefore, a number of immune-related agents have been developed against programmed cell death protein 1 (PD-1), programmed cell death 1 ligand 1 (PD-L1), and cytotoxic T-lymphocyte-associated protein 4 (CTLA-4), which block immune checkpoints and offer prolonged survival in various cancer patients [11,75]. STAT3 hyperactivation facilitates immunosuppression, induced by cancer cells and cells from their microenvironment [76]. STAT3 hyperactivation in cancer cells suppresses the expression of immune-inducing factors, such as interferons (IFNs), pro-inflammatory cytokines (IL-12, tumor necrosis factor-α (TNF-α)), and chemokines (chemokine (CC motif) ligand 5 (CCL5), CXC motif chemokine ligand 10 (CXCL10)), while it increases the expression of alternative cytokines and growth factors, including IL-6, IL-10, transforming growth factor beta (TGF*β*), and VEGF [77]. Furthermore, intense STAT3–NF-κB crosstalk in cancer cells elicits profound immunosuppressive effects. STAT3 hyperactivation in immune cells also mediates inhibition of the innate and adaptive immunity of the TM [78]. Immunosuppression is also achieved through the crosstalk of tumor cells with immune cells, and CAFs form the TM tumor-secreted factors that exert a paracrine function, augmenting STAT3 activation in stroma cells [77].

In CRC tissues and cells, IL-6 triggers STAT3 activation in the TM and suppresses the activation of effector T cells by DCs. This mechanism was demonstrated since tumor-infiltrating CD11b^+^CD11c^+^ show profound expression of IL-6 and decreased ability of T-cell stimulation [79]. Cytoplasmic polyadenylation element-binding protein 3 (CPEB3) exhibits a tumor-suppressive role in CRC, and has been shown to inhibit EMT by interrupting the crosstalk between CRC cells and tumor-associated macrophages, in an IL-6/STAT3-dependent manner [80]. In addition, Th17 cells suppress CD8+ cell migration, which is a prerequisite for the efficacy of immunotherapy, by downregulating CXCR3 expression, through an IL-17A/STAT3 mechanism, in advanced-stage CRC patients [81]. Immunotherapy includes immune checkpoint blockade (ICB) and chimeric antigen receptor T cells (CAR-T). Although the introduction of such agents into the clinic was crucial for a certain number of patients, there are still certain challenges that need to be met in order for immunotherapy to reach wider utility [11]. Combining immunotherapy with targeted agents is an interesting option, and among candidate combination regimens, the combination of immunotherapy and STAT3 blockade may be promising [11]. In CRC cells, PD-L1 expression is triggered by FGFR2, through a JAK/STAT3 signaling pathway, therefore the inhibition of STAT3 could potentially function in synergy with PD-L1 blockade [82]. A recent study demonstrates that a phytical anticancer compound panaxadiol repressed PD-L1 expression, through the dissociation of HIF-1α and STAT3 [83]. Furthermore, resistance to γδT-associated immunotherapy is shown to be conferred via a STAT3/UL16 binding protein 2 (ULBP2) axis [84], indicating that STAT3 targeting may overcome resistance mechanisms to immunotherapy.

## 6. Conclusions

STAT3 has been established as a critical regulator of context-dependent processes in cancer cells and their surrounding stromal cells. Although there are findings that attribute STAT3 either an oncogenic or a tumor-suppressive role in solid tumors and CRC, it has been widely accepted that STAT3 activity mainly lies on STAT3 homodimerization and activation for gene transcription, which regulates proliferation, apoptosis, invasion and metastasis, angiogenesis, and TM immune status in CRC. Therefore, STAT3 inhibition is a promising therapeutic strategy, with a footnote regarding proper context-dependent application. STAT3 targeting should be explored in the future, as part of drug-resistance bypassing mechanisms, as well as in combination regimens that will alleviate the resistance of CRC cells to applied immunotherapy.

## Figures and Tables

**Table 1:** Clinical trials of CRC patients including STAT3 inhibitors (https://clinicaltrials.gov).

Identifier	Status	Title	Condition	Phase	Drug
NCT03647839	Active, not recruiting	Modulation Of The Tumour Microenvironment Using Either Vascular Disrupting Agents or STAT3 Inhibition in Order to Synergise With PD1 Inhibition in Microsatellite Stable, Refractory Colorectal Cancer	Colorectal Cancer Metastatic	II	BBI608
NCT02983578	Active, not recruiting	Danvatirsen and Durvalumab in Treating Patients With Advanced and Refractory Pancreatic, non-small cell Lung Cancer, and Mismatch Repair Deficient Colorectal Cancer	Advanced Colorectal Carcinoma Mismatch Repair Deficiency Refractory Colorectal Carcinoma Stage III Colorectal Cancer AJCC v8 Stage IIIA Colorectal Cancer AJCC v8 Stage IIIB Colorectal Cancer AJCC v8 Stage IIIC Colorectal Cancer AJCC v8 Stage IV Colorectal Cancer AJCC v8 Stage IVA Colorectal Cancer AJCC v8 Stage IVB Colorectal Cancer AJCC v8 Stage IVC Colorectal Cancer AJCC v8	II	Danvatirsen
NCT03195699	Recruiting	Oral STAT3 Inhibitor, TTI-101, in Patients With Advanced Cancers	Colorectal Cancer Advanced Cancer	I	TTI-101
NCT03522649	Recruiting	A Phase III Clinical Study of Napabucasin (GB201) Plus FOLFIRI in Adult Patients With Metastatic Colorectal Cancer	Previously Treated Metastatic Colorectal Cancer	III	BBI608
NCT02753127	Completed	A Study of Napabucasin (BBI-608) in Combination With FOLFIRI in Adult Patients With Previously Treated Metastatic Colorectal Cancer (CanStem303C)	Colorectal cancer	III	BBI608
NCT01776307	Completed	A Study of BBI608 in Adult Patients With Advanced Colorectal Cancer	Colorectal Cancer	II	BBI608
NCT03522649	Recruiting	A Phase III Clinical Study of Napabucasin (GB201) Plus FOLFIRI in Adult Patients With Metastatic Colorectal Cancer	Previously Treated Metastatic Colorectal Cancer	III	BBI608
NCT02641873	Completed	A Study of BBI608 Administrated With FOLFIRI + Bevacizumab in Adult Patients With Metastatic Colorectal Cancer	Metastatic Colorectal Cancer	I	BBI608
NCT03647839	Active, not recruiting	Modulation Of The Tumour Microenvironment Using Either Vascular Disrupting Agents or STAT3 Inhibition in Order to Synergise With PD1 Inhibition in Microsatellite Stable, Refractory Colorectal Cancer (MODULATE)	Colorectal Cancer Metastatic	II	BBI608
NCT01830621	Completed	BBI608 and Best Supportive Care vs. Placebo and Best Supportive Care in Pretreated Advanced Colorectal Carcinoma	Colorectal Carcinoma	III	BBI608
